# Hyaluronic-Acid Nanocapsules with Plant Extracts: Characterization and Antimicrobial Activity Against Skin Microbiota

**DOI:** 10.3390/ma19071288

**Published:** 2026-03-24

**Authors:** Anna Lenart-Boroń, Anna Ratajewicz, Natalia Czernecka-Borchowiec, Anna Kopacz, Zofia Schejbal, Gohar Khachatryan, Karen Khachatryan, Magdalena Krystyjan, Klaudia Bulanda, Klaudia Stankiewicz

**Affiliations:** 1 Department of Microbiology and Biomonitoring, University of Agriculture in Kraków, Mickiewicza Ave. 24/28, 30-059 Kraków, Poland; 2 Department of Zoology, Institute of Biology and Earth Sciences, Faculty of Exact and Natural Sciences of the University of the National Education Commission, Podchorążych 2, 30-084 Kraków, Poland; anna.ratajewicz@student.urk.edu.pl; 3 Scientific Circle of Biotechnologists, University of Agriculture in Kraków, 31-425 Kraków, Poland; natalia.czernecka2@student.urk.edu.pl (N.C.-B.); anna.kopacz@student.urk.edu.pl (A.K.);; 4 Department of Food Analysis and Quality Assessment, Faculty of Food Technology, University of Agriculture in Kraków, Al. Mickiewicza 21, 31-120 Kraków, Poland; gohar.khachatryan@urk.edu.pl; 5 Laboratory of Nanotechnology and Nanomaterials, Faculty of Food Technology, University of Agriculture in Kraków, Al. Mickiewicza 21, 31-120 Kraków, Poland; karen.khachatryan@urk.edu.pl; 6 Department of Carbohydrates Technology and Cereal Processing, Faculty of Food Technology, University of Agriculture in Kraków, Al. Mickiewicza 21, 31-120 Kraków, Poland; magdalena.krystyjan@urk.edu.pl; 7 Department of Forest Ecosystems Protection, Faculty of Forestry, University of Agriculture in Kraków, 29 Listopada Ave. 46, 31-425 Kraków, Poland; 8 Department of Pharmaceutical Microbiology, Faculty of Pharmacy, Jagiellonian University Medical College, Medyczna 9 Street, 30-688 Kraków, Poland

**Keywords:** antimicrobial activity, *Arnica montana*, biocompatible materials, hyaluronic acid, MALDI-TOF, plant extracts, skin microbiota, topical formulations

## Abstract

**Highlights:**

**What are the main findings?**

- HA nanocapsules with plant extracts were successfully synthesized.
- Nanocomposites showed stable physicochemical and structural properties.
- Extract type determined distinct antimicrobial activity profiles.

**What is the implication of the main finding?**

- Nanocapsules are suitable carriers for topical bioactive delivery.
- Formulations can be tailored for cosmetic or dermatological uses.
- Extract-specific activity enables targeted skincare applications.

**Abstract:**

Hyaluronic acid (HA)–based nanocapsules containing plant-derived bioactives are promising formulations for dermatological applications. In this study, nanocapsules containing extracts of *Arnica montana*, *Calendula officinalis* and *Aesculus hippocastanum* were synthesized and their structural and functional properties were characterized. Scanning electron microscopy confirmed the formation of spherical nanostructures with uniform morphology, while rheological analyses demonstrated stable viscoelastic behavior suitable for topical application. Their antimicrobial potential was assessed on microorganisms isolated from multiple regions of healthy human skin and opportunistic pathogens. A diverse panel of approx. 100 bacterial and fungal isolates was identified using MALDI-TOF MS. The antimicrobial activity of formulations was compared with commonly used disinfectants: H_2_O_2_, octenidine, isopropanol and topical ophthalmic antiseptic. Arnica-based formulations showed the strongest inhibitory effect against both Gram-positive and Gram-negative bacteria, whereas chestnut extract demonstrated selective activity against *Candida* spp. Calendula-based formulations exhibited limited antimicrobial activity. These findings demonstrate that plant-extract-loaded HA nanocapsules exhibit selective antimicrobial properties dependent on extract type and microbial group, supporting their potential as multifunctional components of future dermatological formulations.

## 1. Introduction

Human skin forms the primary barrier of the human body against the external environment and therefore it is continuously exposed to a variety of challenges. Its surface is naturally colonized by a diverse community of microorganism that, under physiological conditions, coexist harmlessly with their host and contribute to maintaining cutaneous homeostasis [1]. This natural skin microbiota acts as an important first line of defense by limiting the growth and invasion of potentially harmful species. However, commensal microorganisms inhabiting the human skin can also include opportunistic pathogens, such as *Staphylococcus aureus*, which can cause infections in cases favorable for such an infection [2]. Among such conditions, epidermis continuity disruption is among the frequent factors contributing to *S. aureus* infections. Even minor injuries, needle punctures, or irritation following cosmetic or aesthetic procedures may facilitate microbial penetration and increase the risk of local infections [3,4].

To support skin regeneration and alleviate inflammation after disruptions, a variety of topical preparations, based on plant extracts, are commonly used as one of the first therapeutic resources [5]. These include ointments or gels containing extracts of plants with vasoprotective, anti-inflammatory and/or regenerative properties, such as arnica (*Arnica montana*), horse chesnut (*Aesculus hippocastanum*) or calendula (*Calendula officinalis*) [5,6,7]. Plant-derived extracts are widely incorporated into cosmetic and pharmaceutical formulations due to their long history of use, favorable safety profiles, and multifunctional biological activity. The active compounds of these extracts include a variety of secondary metabolites, such as: sesquiterpenes, terpenes, carotenoids, flavonoids, tannins coumarins, essential oils and phenolic compounds [5,6,8].

Products containing these extracts are frequently used by individuals prone to bruising, by children and athletes, and by people undergoing aesthetic or cosmetic treatments. Most commercially available formulations rely on alcohol-based extracts combined with petrolatum or glycerin. Although these substances are well tolerated, they do not always effectively penetrate the *stratum corneum*, which may result in varying delivery of active compounds to deeper skin layers [9]. Importantly, the depth of compound penetration into the skin layers strongly depends on the delivery system [10]. Extended and frequent use of alcohol-based hand sanitizers is increasingly recognized as a factor contributing to skin barrier impairment. Alcohol dissolves intracellular lipids, dehydrates the *stratum corneum* and disrupts natural moisturizing factors, as a result compromising the integrity of the lipid barrier [11]. These changes may lead to irritant contact dermatitis, erythema, fissuring, and increased transepidermal water loss. Moreover, excessive sanitizer use can disturb the skin microbiome by eliminating protective commensal species, a process associated with dysbiosis-related conditions such as eczema, inflammation, or susceptibility to secondary infections [12]. Recent discussions have also raised concerns that repeated exposure to sanitizer products contaminated with additives such as benzene, toluene and styrene may increase the risks of cancer [13].

The popularity of plant extract-based preparations has grown recently, mainly due to increasing demand for formulations based on natural substances, the expanding use of aesthetic medicine procedures that frequently result in bruising or transient barrier disruption, and the demand for gentle, well-tolerated preparations suitable for children and individuals with sensitive skin. In recent years, several studies have evaluated the antimicrobial properties of plant-derived topical formulations or advanced biopolymer-based delivery systems, highlighting their potential relevance for preventing microbial colonization of compromised skin [14,15]. For example, recent work has demonstrated antimicrobial effects of newly developed glycerol–silicone adhesive capable of releasing octenidine [16]. However, despite these advances, there is still a lack of studies assessing the antimicrobial activity of hyaluronic acid-based carriers loaded with plant extracts against a broad and clinically relevant panel of skin microbiota. This gap provides the rationale for our study.

With the growing need for topical formulations that combine the biological activity of plant extracts with improved skin penetration and therapeutic potential, this study aimed to address this need by developing innovative bionanocomposites in which extracts of arnica, calendula, and horse chestnut are encapsulated within hyaluronic acid-based nanostructures. Hyaluronic acid is a biocompatible polymer, known to modulate inflammation and promote tissue repair [17]. Encapsulation within hyaluronic acid is expected to enhance the stability, penetration, and controlled release of the active compounds, potentially improving their clinical utility.

A key step in the development of such formulations is the evaluation of their antimicrobial activity, particularly given the central role of skin-associated microorganisms in both health and disease. Understanding how different plant extracts interact with representative members of the skin microbiota—including commensals, opportunistic pathogens, and yeasts—is essential for determining their suitability for various dermatological applications. In view of the above, the present study aimed to develop and characterize innovative hyaluronic acid-based nanocomposites incorporating extracts of *Arnica montana*, *Aesculus hippocastanum* and *Calendula officinalis*. Specifically, the objectives were to: (i) formulate stable nanocomposites through the encapsulation of plant-derived bioactive compounds within a hyaluronic acid matrix, (ii) characterize their structural, physicochemical, optical, rheological, and textural properties, and (iii) evaluate and compare their antimicrobial activity against a broad panel of microorganisms representative of the human skin microbiota, including commensal species, opportunistic pathogens and yeasts. Particular emphasis was placed on assessing extract-dependent differences in antimicrobial efficacy and selectivity, in order to identify formulations with potential applicability in dermatological and cosmetic products intended to support microbial homeostasis or prevent skin colonization by opportunistic pathogens.

## 2. Materials and Methods

### 2.1. Materials

The following chemical reagents and materials were used: high molecular weight hyaluronic acid (Aquajuv CT) and molecular weight 0.8–1.0 MDa, (Organic Zing, New Delhi, India); dried flowers of *Arnica montana* and *Calendula officinalis* (PERSEA Sp. z o.o., Warsaw, Poland); dried flowers of *Aesculus hippocastanum* (Dary Podlasia, Bielsk Podlaski, Poland); and deionised water (demineraliser Polwater DL3-150, Labopol-Polwater, Kraków, Poland).

### 2.2. Preparation of Nanocomposites

#### 2.2.1. Plant Materials and Extraction

Dried flowers of *Arnica montana* and *Calendula officinalis* were purchased from PERSEA Sp. z o.o. (Warsaw, Poland), and dried flowers of *Aesculus hippocastanum* were obtained from Dary Podlasia (Bielsk Podlaski, Poland). All plant materials were stored in a dry, dark place at room temperature until use. Plant extracts were prepared by Soxhlet extraction. Briefly, 30.0 g of ground plant material (flowers) were extracted with 250 mL of methanol (analytical grade, Pol-Aura, Morąg, Poland) for 5 h. After extraction, the solvent was removed under reduced pressure using a rotary evaporator (Heidolph Instruments GmbH & Co. KG, Schwabach, Germany) at 40 °C, yielding dry extracts. The extraction yield was calculated as:

$$Y\;\left(\%\right)=\;\frac{m_{1}}{m_{2}}\times 100\%$$

where *m*_1_ is the mass of the dry extract (g) and *m*_2_ is the mass of the raw material on a dry matter basis (g).

The extraction yields varied depending on the botanical origin: *A. montana* 49.02%, *C. officinalis* 44.25%, and *A. hippocastanum* 42.38% (Table 1). The dried extracts were stored in sealed containers protected from light at 4 °C until formulation.

#### 2.2.2. Preparation of Hyaluronic Acid Gel

A total of 5.0 g of high molecular weight hyaluronic acid was slowly added to 495.0 g of deionised water. The resulting suspension was kept at 40 °C for 2 h with vigorous stirring (700 rpm; Heidolph RZR 2020, Heidolph Instruments GmbH & Co. KG, Schwabach, Germany). Subsequently, the sample was stirred at 23 °C for 24 h to ensure complete hydration.

#### 2.2.3. Preparation of Nanoemulsions

To 5.0 g of dry plant extract (obtained as described in Section 2.2.1), 5.0 g of deionised water and 5.0 g of pomegranate seed oil were added. Each mixture was homogenized using an ultrasonic processor (15 min, 20 kHz; Sonopuls HD 4200, Bandelin, Berlin, Germany) to obtain three distinct nanoemulsions, designated AM, AH, and CO (as in *Arnica montana*, *Aesculus hippocastanum* and *Calendula officinalis*).

#### 2.2.4. Preparation of the Control (C) Sample (H0)

To 100 g of the hyaluronic acid (HA) gel, 10.0 g of deionised water was added gradually under continuous homogenisation (12,000 rpm, Polytron PT 2500E, Kinematica AG, Malters, Switzerland) until a homogeneous gel was obtained. This sample served as the control (H0).

#### 2.2.5. Preparation of Nanocomposite Formulations

a. Formulation Series I: To 100 g of the HA gel, 5.0 g of the respective nanoemulsion (AM, AH, or CO) and 5.0 g of deionised water were added gradually under continuous homogenisation (12,000 rpm, Polytron PT 2500E, Kinematica AG, Malters, Switzerland) until a uniform emulsion was obtained. The resulting samples were designated AM-I, AH-I, and CO-I.

b. Formulation Series II: To 100 g of the HA gel, 10.0 g of the respective nanoemulsion (AM, AH, or CO) was added gradually under continuous homogenisation (12,000 rpm, Polytron PT 2500E, Kinematica AG, Malters, Switzerland) until a uniform emulsion was achieved. The resulting samples were designated AM-II, AH-II, and CO-II.

#### 2.2.6. Sample Preparation for Analysis

For further analyses, 30 g of each prepared gel sample was poured into Petri dishes and dried at 40 °C for approximately 24 h. The dried films were subsequently used for characterization by scanning electron microscopy (SEM) and Fourier Transform Infrared (FTIR) spectroscopy.

### 2.3. Scanning Electron Microscopy

A JEOL 7550 scanning electron microscope (Akishima, Tokyo, Japan) equipped with secondary electron detection was used to characterize and visualize the size and morphology of the nano/microcapsules developed in our experiments. The instrument operated at 15 kV with a 1.0 nm spot size. For optimal imaging conditions, the samples were metallized with a 20 nm chromium layer using a K575X Turbo Sputter Coater (Fedelco, Madrid, Spain) to enhance sample conductivity.

### 2.4. FTIR Spectroscopy

FTIR spectra of the prepared films were recorded with a Mattson 3000 FT-IR spectrophotometer (Mattson Unicam, Madison, WI, USA) equipped with a ReFractance 30SPEC 30-angle reflectance overlay and MIRacle ATR module (PIKE Technologies Inc., Madison, WI, USA). The spectra were acquired at a resolution of 4 cm, in the infrared region of 4000–400 cm^−1^. The data were subsequently processed by applying baseline correction (automatic polynomial fitting), ATR absorption adjustment and vector normalization. All processing steps were carried out using Omnic 9 software (v9.12.1002, Thermo Fisher Scientific).

### 2.5. UV-Vis Spectroscopy

Spectral measurements were performed with a SHIMADZU TCC-260 scanning spectrophotometer (SHIMADZU, Kyoto, Japan) over the 200–700 nm wavelength range. The 8 × 40 mm^2^ film strips were placed in a 10 mL quartz cuvette (10 mm thick quartz cells), while an empty cuvette served as the reference.

### 2.6. Color Measurement

Color evaluation was carried out with a CM-3500d spectrophotometer (Konica Minolta Inc., Tokyo, Japan) operating with a 30 mm measurement aperture, under D65 illumination and a 10° standard observer. Color parameters were determined in the CIELAB system (L*, a*, b*), with L* representing lightness (0–100), a* indicating the green–red axis (− green, + red), and b* corresponding to the blue–yellow axis (− blue, + yellow). Each sample was measured in five replicates, and the results were expressed as means of these replicates. Using the obtained L*, a*, and b* values, chroma (C*), hue angle (h°), and total color difference (ΔE*) were computed [18]. Chroma (C*) represents color saturation, where higher values correspond to more intense colors:

$$C^{*}=\;\sqrt{{a^{*}}^{2}+{b^{*}}^{2}}$$

The hue angle (h°) defines the color position in the CIELAB space, with 0°, 90°, 180°, and 270° corresponding to red, yellow, green, and blue, respectively:

$$h^{*}={tan}^{-1}\left(\frac{b^{*}}{a^{*}}\right)$$

The total color difference (ΔE*) quantifies the color change relative to the control sample [19]:

$${∆E}^{*}=\;\sqrt{{∆a}^{*2}+{∆b}^{*2}+∆L^{*2}}$$

### 2.7. Rheological Measurement

Rheological measurements were carried out using a rotary rheometer (RheoStress RS 6000, Thermo Scientific, Karlsruhe, Germany) equipped with a plate–plate measuring system. The analyses were performed in duplicates at a controlled temperature of 25.0 ± 0.1 °C. An oscillatory stress sweep was conducted over a stress range of 0.1–300 Pa, applied in 40 logarithmically spaced steps at a constant frequency of 1 Hz. A frequency sweep test was also performed by increasing the frequency from 0.1 to 10 Hz at a constant stress of 0.1 Pa, within the linear viscoelastic region [20].

### 2.8. Texture Analyses

The textural properties of the samples were determined using a TA.XTplus texture analyser (Stable Micro Systems Ltd., Godalming, UK), according to Krystyjan et al. [21], with minor modifications. The penetration test was performed using a cylindrical probe P/20 (20 mm diameter) at a test speed of 1 mm/s. Samples placed in 55 mm diameter containers were subjected to compression to a depth of 20 mm under a constant load of 2 g at 25.0 ± 0.1 °C. Based on the obtained force–distance curves, rupture strength (N) and adhesiveness (N·s) were calculated. Rupture strength was defined as the maximum force recorded during probe penetration, corresponding to the point of gel structure failure. Adhesiveness was defined as the work required to detach the probe from the sample surface, corresponding to the negative area of the force–time curve during probe withdrawal.

### 2.9. Antimicrobial Activity Assays

Microorganisms for the study were isolated from both healthy individuals, representing the commensal skin microbiota, and from patients presenting with various dermatological conditions, including inflammatory, infectious and barrier-disruption disorders.

The samples were collected using sterile cotton swabs, gently rolled over a defined skin area and immediately transported to the laboratory for further processing. Then, swabs were streaked onto selective and non-selective media: Columbia Agar with Blood (Thermo Fisher Scientific, Waltham, MA, USA), Tryptone Bile X-glucuronide agar (Biomaxima, Lublin, Poland), Slanetz-Bartley agar (Biomaxima, Lublin, Poland), UTI Chromagar (Thermo Fisher Scientific, Waltham, MA, USA), Baird-Parker agar (Thermo Fisher Scientific, Waltham, MA, USA) and Sabouraud agar (Biomaxima, Lublin, Poland), and incubated under appropriate conditions depending on the expected microbial group (36 ± 1 °C for bacteria and 30 °C for yeasts). Distinct colonies grown on the culture media were then subcultured to obtain pure isolates.

Species identification of the isolates was carried out using matrix-assisted laser desorption/ionization time-of-flight mass spectrometry (MALDI-OF MS) (Bruker, Billerica, MA, USA). Identification scores were interpreted according to the manufacturer’s criteria. The final microbiological panel included a representation of human commensal skin microbiota, opportunistic pathogens as well as true pathogens, i.e., Gram-positive bacteria, Gram-negative bacteria and yeasts.

The antimicrobial activity of plant-derived emulsions and reference antiseptic preparations was evaluated using diffusion-based methods adapted to the physical properties of each formulation.

Emulsions containing *Arnica montana*, *Calendula officinalis* and *Aesculus hippocastanum* extracts were tested with hyaluronan-based emulsion as a control. Due to high viscosity and density of these emulsions, their antimicrobial activity was assessed using the agar well-diffusion method. For these tests, standardized microbial suspensions (0.5 McFarland) were spread onto Mueller-Hinton agar (Biomaxima, Lublin, Poland) (bacteria) or Sabouraud dextrose agar (Biomaxima, Lublin, Poland) (yeasts). Wells of 5 mm in diameter were cut in the agar using a sterile corkborer and filled with 100 µL of each emulsion.

For comparison, commercially available antimicrobial products were included, i.e., stye-eye lipogel (containing terpinen-4-ol as the active ingredient), surface disinfectant (containing 60 g of propan-2-ol and 15 g of ethanol in 100 g), and skin disinfectant (containing 0.10 g of octenidine dihydrochloride and 2.00 g of phenoxyethanol in 100 g). Because these preparations were fluid and compatible with filter disks, they were evaluated using the disk diffusion method. Blank sterile disks were loaded with 15 µL of each preparation and placed onto inoculated agar plates. Such prepared plates were incubated overnight at room temperature to allow for the nanocapsule opening and then were transferred to incubators (36 ± 1 °C for bacteria and 30 °C for yeasts) for 24 h. After incubation, zones of microbial growth inhibition were measured in milimeters (mm).

Antimicrobial testing was conducted in two phases. First, a preliminary screening of all emulsions and antiseptics was tested against a diverse panel of 57 microbial isolates (including 32 Gram-positive bacteria, 13 Gram-negative bacteria and 12 yeasts). Based on these preliminary results, emulsions with two concentrations of arnica extracts were selected for further testing. These tests were conducted using a total of 110 isolates, including 76 Gram-positive bacteria, 19 Gram-negative bacteria and 15 yeasts.

### 2.10. Statistical Analyses

Statistical analyses were performed using Statistica v. 13 (TIBCO, Palo Alto, CA, USA). One-way analysis of variance, followed by a post hoc Fisher’s test (*p* < 0.05) was conducted to assess the significance of differences between the examined groups of formulations.

## 3. Results and Discussion

### 3.1. Scanning Electron Microscopy-Based Determination of the Obtained Nanocapsules

**Figure 1:**
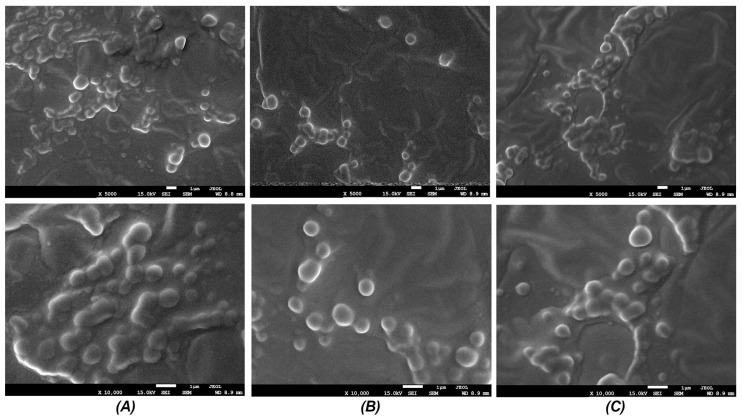
Scanning electron microscopy (SEM) images of the developed nanocomposite films: (**A**) *Arnica montana* (AM); (**B**) *Aesculus hippocastanum* (AH); and (**C**) *Calendula officinalis* (CO). The images reveal spherical nano/microcapsules embedded within the hyaluronic acid matrix.

The SEM analysis revealed the formation of spherical nano/microcapsules in all tested formulations (AM, AH, CO) (Figure 1). The particles exhibited a predominantly regular, round morphology with a smooth surface, indicating successful encapsulation within the hyaluronic acid (HA) gel matrix. The capsule size distribution was uniform in each sample, approximately 500–1500 nm.

Significant differences in capsule size and morphology were observed depending on the plant extract used. The capsules containing *Arnica montana* (AM) extract displayed the smallest average diameter and the most uniform spherical shape. Formulations with *Aesculus hippocastanum* (AH) showed slightly larger capsules with a similarly smooth surface. In contrast, samples containing *Calendula officinalis* (CO) extract presented capsules of comparable size to AH but occasionally exhibited a less uniform surface or minor signs of coalescence, which could be attributed to the specific interfacial properties of this extract.

The SEM results confirm the successful formation of a nano/microcapsule system within the hyaluronic acid gel, achieved through the described sequential process of pre-emulsification via sonication and subsequent incorporation into the HA matrix under homogenization.

The formation mechanism can be explained as follows: the sonication of the aqueous extract solution with pomegranate seed oil created a fine pre-emulsion with submicron oil droplets. Upon introduction into the high molecular weight hyaluronic acid gel under shear homogenization, the long-chain HA polymers likely act as both an emulsifying and a matrix-forming agent. The polymeric chains adsorb at the oil–water interface, forming a stabilizing layer around the droplets. During gel setting, this interfacial HA layer, along with the bulk HA gel, solidifies into a matrix, entrapping the oil droplets containing the plant extract, thus forming a core (oil + extract)–shell/matrix (HA) structure. The spherical morphology observed in SEM is characteristic of such emulsion-templated systems and suggests effective stabilization by the HA [22,23,24].

The observed differences in capsule characteristics between the plant extracts (AM, AH, CO) are noteworthy. These variations can be attributed to the distinct chemical compositions of each extract, which influence the interfacial tension at the oil–water boundary during pre-emulsification and subsequently affect the interaction with the HA polymer. Components such as saponins (present in *Aesculus hippocastanum*), flavonoids, or other surface-active molecules in the extracts [25] may play a co-emulsifying role, impacting the final droplet size and the stability of the interface, leading to the morphological differences captured by SEM. The excellent uniformity of AM-based capsules suggests its constituents are particularly compatible with this encapsulation process.

SEM analysis visually confirms the successful encapsulation of plant extracts into a hyaluronic acid-based delivery system. The method yields spherical capsules whose precise size and surface morphology are influenced by the specific botanical extract used.

A limitation of the present study is that capsule size was assessed only from SEM images without complementary dynamic light scattering (DLS) measurements or quantitative image analysis. The reported size range (500–1500 nm) is therefore based on qualitative visual observations of multiple micrographs. Future work will include DLS analysis to provide a more detailed description of the hydrodynamic size distribution in aqueous dispersion, as well as quantitative image analysis (e.g., using ImageJ software, v. 1.54p) to obtain mean particle diameters, standard deviations and polydispersity indices for each formulation.

### 3.2. FTIR Spectroscopy

Fourier Transform Infrared (FTIR) spectroscopy was used to analyze the chemical composition and identify potential interactions within the prepared nanocomposites containing extracts of *Arnica montana* (AM), *Aesculus hippocastanum* (AH), and *Calendula officinalis* (CO) embedded in a hyaluronic acid (HA) gel matrix with pomegranate seed oil. The analysis of key absorption bands confirms the successful formation of composite materials and reveals distinct spectral profiles influenced by the botanical extract used.

**Figure 2:**
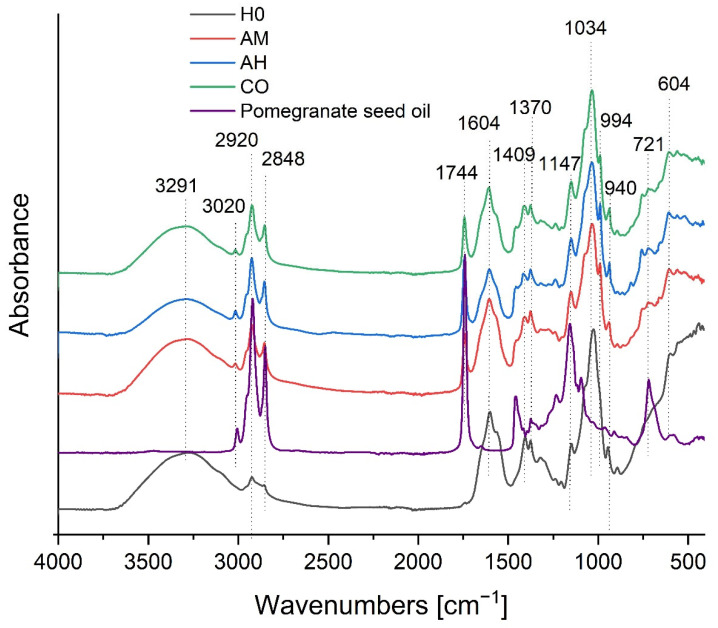
FTIR spectra of the control hyaluronic acid gel (H0), pomegranate seed oil, and the nanocomposite formulations containing plant extracts: *Arnica montana* (AM), *Aesculus hippocastanum* (AH), and *Calendula officinalis* (CO). Key absorption bands characteristic of lipids (1744, 2920, 2848 cm^−1^), punicic acid markers (3020, 994 cm^−1^), and the polysaccharide matrix are highlighted.

The most prominent observation is the significant spectral shift between the control HA gel (H0) and all nanocomposite formulations. The pure HA gel exhibits the most intense band at approximately 3291 cm^−1^, characteristic of O–H stretching vibrations from the polysaccharide matrix and bound water. In the nanocomposite samples (AM, AH, CO), the relative intensity of this hydroxyl band is markedly reduced. Concurrently, a strong and well-defined peak emerges at 1744 cm^−1^, which is the signature absorption band for the C=O stretching vibration of ester carbonyl groups in triglycerides, unequivocally confirming the incorporation of pomegranate seed oil into the system (Figure 2).

Furthermore, the spectra of the nanocomposites and the oil control display specific markers characteristic of punicic acid (conjugated linolenic acid), the predominant fatty acid in pomegranate seed oil. A distinct weak band at ~3020 cm^−1^ (=C–H stretching) and a sharp, intense band at ~994 cm^−1^ (deformation vibration of conjugated cis-trans double bonds) are clearly visible. The presence of these specific bands confirms that the chemical integrity of the bioactive oil phase was preserved during the sonication and homogenisation processes [26].

The spectra are not simple superimpositions of the individual components, indicating chemical or physical interactions within the composite. Notably, the bands associated with HA’s functional groups, such as the amide I band (~1604 cm^−1^) and the carboxylate band (~1409 cm^−1^), show alterations in their relative intensities and profiles in the nanocomposites. These changes suggest the occurrence of intermolecular interactions—likely hydrogen bonding or electrostatic forces—between the polar groups of hyaluronic acid and the various phytochemicals (e.g., phenolic acids, flavonoids, saponins) present in the plant extracts. Such interactions are crucial for stabilizing the nanocomposite structure [27].

Differences among the composites containing different plant extracts are discernible, particularly in the fingerprint region. The sample formulated with *Calendula officinalis* (CO) demonstrates the most pronounced intensity at 1034 cm^−1^, corresponding to the C–O–C stretching vibrations of the polysaccharide backbone. This suggests that the calendula extract may induce a distinct spatial organization of the HA matrix or interact more strongly with the polymer chains compared to the AM and AH extracts. While all composites show successful lipid incorporation (bands at 2920, 2848, and 1744 cm^−1^), the spectral nuances in the 900–1200 cm^−1^ region highlight the influence of the specific botanical extract’s composition on the final material structure.

In summary, FTIR analysis provides strong chemical evidence for the successful formation of nanocomposites incorporating plant extracts. The results confirm the integration of pomegranate seed oil within the hyaluronic acid gel and indicate the presence of intermolecular interactions that contribute to the stabilization of the composite structure. The observed differences among the AM, AH, and CO samples highlight the significant influence of the chemical composition of individual botanical extracts on the final properties of the nanocomposite, which may affect the stability of the active compounds. These findings correlate well with, and are supported by, the morphological observations obtained using scanning electron microscopy (SEM).

Similar shifts in the O–H stretching region, the appearance of intense lipid-associated carbonyl bands at ~1744 cm^−1^, and alterations in polysaccharide backbone vibrations have been reported for other HA-containing or polysaccharide-based nanocomposites incorporating vegetable oils, essential oils or plant extracts [22,23,24,27,28]. In those studies, hydrogen bonding and electrostatic interactions between the polar groups of the biopolymer matrix and phenolic or terpenoid phytochemicals were shown to stabilize the composite structure and preserve the chemical integrity of encapsulated bioactives during processing. These literature data are fully consistent with the FTIR signatures observed in our HA-based nanocapsules and support the conclusion that intermolecular interactions contribute to the structural stability of the present formulations.

### 3.3. UV-VIS

The optical properties of the formulated nanocomposite gels were characterized using Ultraviolet–Visible (UV-Vis) spectroscopy, focusing on the spectral region between 200 and 700 nm. The absorbance profiles of the composites containing extracts of *Arnica montana* (AM), *Aesculus hippocastanum* (AH), and *Calendula officinalis* (CO) were compared with the control hyaluronic acid (HA) gel.

**Figure 3:**
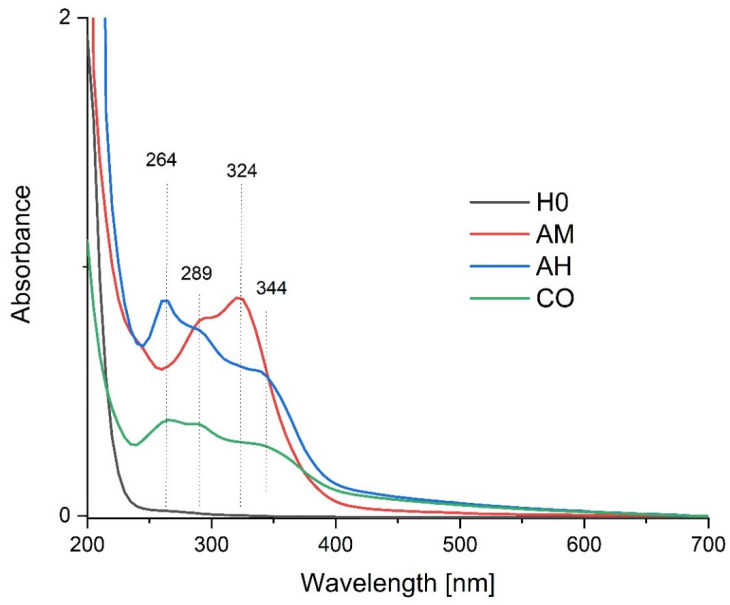
UV-Vis absorption spectra of the control hyaluronic acid gel (H0) and the nanocomposite films containing *Arnica montana* (AM), *Aesculus hippocastanum* (AH), and *Calendula officinalis* (CO). Distinct absorption maxima at 264, 289, 324, and 344 nm correspond to the specific phytochemical profiles (flavonoids, phenolic acids) of the encapsulated extracts.

The UV-Vis spectrum (Figure 3) of the pure HA gel is largely featureless in the analyzed region, showing only a strong, broad absorption below 220 nm attributable to the polysaccharide backbone, confirming the absence of significant UV-absorbing chromophores in the base gel matrix. In marked contrast, the spectra of all three nanocomposites exhibit a dramatic increase in absorbance across the UV range, with well-defined maxima that correspond to the electronic transitions of specific phytochemical classes.

The specific spectral signatures correlate directly with the botanical source of the extract:

- Arnica montana (AM): This formulation displays prominent absorption maxima at 324 nm and 344 nm. These peaks are characteristic of Band I (cinnamoyl system) electronic transitions, typical of flavonoids such as the quercetin derivatives and phenolic acids abundant in arnica [28,29].
- Aesculus hippocastanum (AH): The spectrum is dominated by a sharp, intense peak at ~264 nm. This absorption corresponds to the Band II (benzoyl system) transitions, likely attributable to coumarin glycosides (e.g., esculin, fraxin) and phenolic acids, which are the primary bioactive constituents of horse chestnut [30].
- Calendula officinalis (CO): While exhibiting the UV peaks characteristic of phenolics, the CO spectrum is distinguished by a broader absorption profile that extends into the visible region (>400 nm). This elevated baseline and “tailing” effect are indicative of the presence of carotenoids (e.g., lutein, beta-carotene), which are well-known chromophores in calendula flowers [28,31].

The presence of these distinct peaks confirms that the extraction and subsequent nanoencapsulation processes effectively preserved the bioactive constituents. The nanocomposites are not merely physical mixtures but functional materials where the phytochemical payload dominates the optical properties. The intrinsic UV absorption capability imparted by these natural phytochemicals suggests that the nanocomposites could offer supplementary UV-filtering effects in topical applications, leveraging the multifunctional nature of the encapsulated plant extracts [31].

### 3.4. Color Parameters of Emulsions

Table 2 shows the values of the L* parameter, describing color lightness in the CIE Lab system. The control sample (pure hyaluronan gel) exhibited the highest lightness. The incorporation of plant extracts into the hyaluronan gel resulted in a noticeable darkening of the composites. The largest decrease in L* compared with the control was observed for the sample containing arnica extract (AM), whereas the remaining samples showed slightly higher (~65–67) and comparable values. The results also indicate a clear influence of extract concentration on the color parameters of the biocomposites (Table 2 and Table 3).

In most samples, a predominance of red over green and yellow over blue was observed (a* > 0, b* > 0). Only sample AM I showed a predominance of green over red (a* < 0). The green coloration of arnica is mainly related to the presence of chlorophyll, whose content is typically higher in leaves than in flowers, where carotenoids and flavonoids have also been identified [32]. The higher extract content in AM II shifted the color towards yellow–brown tones, likely due to the presence of carotenoids and flavonoids in arnica extract, which reduced the relative contribution of green tones observed in AMI (Table 2). Samples COII and AHII showed the highest contribution of pigments responsible for red coloration. Color saturation (C*) in extract-enriched samples was markedly higher than in the control, and color intensity increased with extract concentration in the gel. The calendula extract exhibited the greatest color intensity, mainly due to carotenoids, particularly lutein and its derivatives, and to a lesser extent flavonoids and chlorophyll. The total carotenoid content in calendula flowers depends on plant genotype, flower color, cultivation conditions, and harvest time [33,34]. In horse chestnut, the characteristic brown color is associated with phenolic compounds such as tannins, flavonoids, and coumarins. Chlorophyll and typical flower pigments (anthocyanins) are not present in horse chestnut seeds; therefore, their color is primarily determined by polyphenols and tannins [35,36]. The total color difference between the samples and the control indicates clearly perceptible differences in color between the analyzed samples and the control (ΔE* > 5). The color of the emulsions will directly influence the perceived color of the resulting wound dressings. More intense and warmer shades (yellow) may be associated with greater biological activity or a higher content of active ingredients, while green tones may suggest naturalness. A higher color saturation will enhance the visual clarity and distinctiveness of the dressings, potentially influencing both their attractiveness and the user’s subjective perception of efficacy.

### 3.5. Rheological Properties of Emulsions

**Figure 4:**
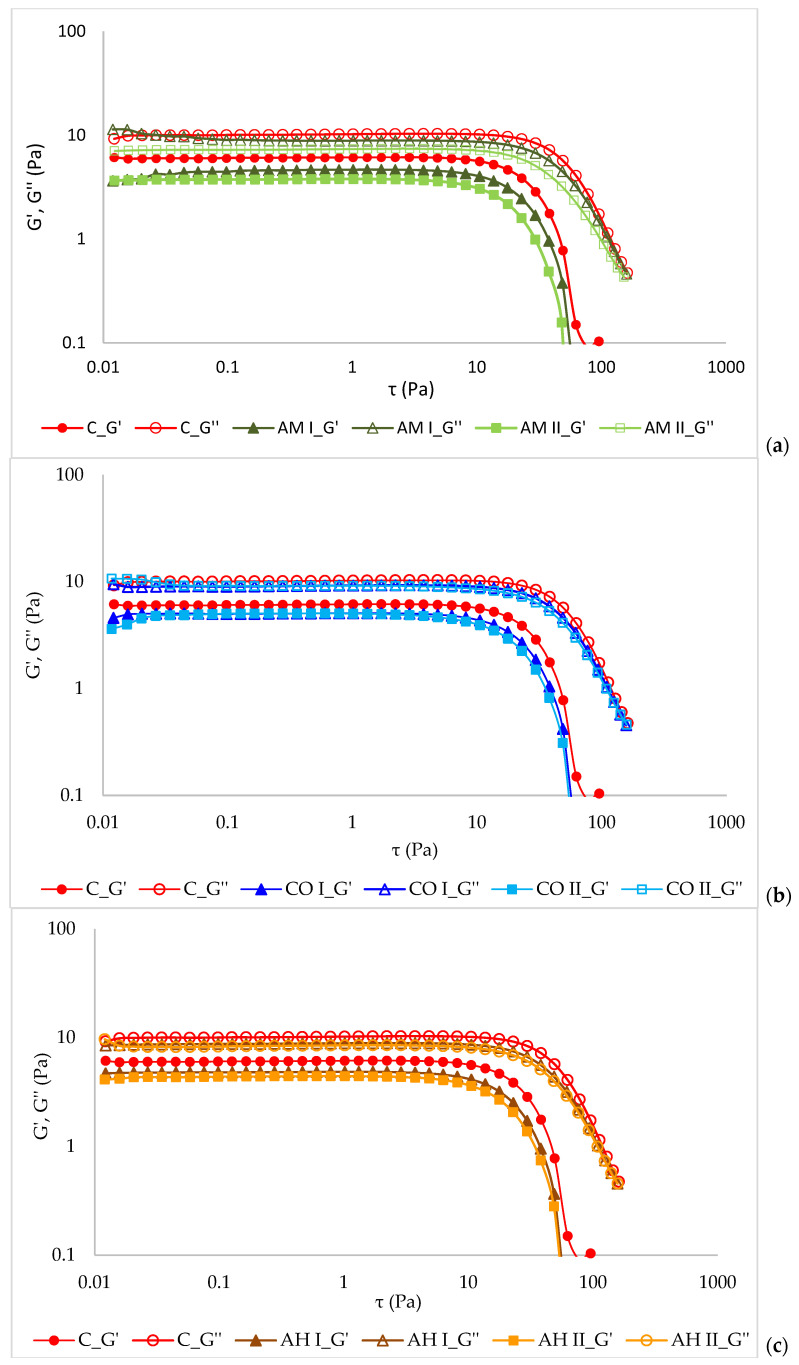
Values of storage and loss moduli (G′ and G″) measured in stress sweep test of (**a**) AM, (**b**) CO and (**c**) AH.

Figure 4 presents the strain-sweep results for the storage (G′) and loss (G″) moduli of the emulsions. This analysis was performed to determine the linear viscoelastic region (LVR) and to assess the structural stability of the systems [37]. The LVR corresponds to the range of deformation in which both moduli remain constant, indicating that the internal structure of the material is not disrupted. A wider LVR is generally associated with greater structural strength and stability of the material. The storage modulus (G′) reflects the elastic response of the system, whereas the loss modulus (G″) describes its viscous behavior [38]. The analyzed nanoemulsions exhibited similar rheological characteristics, including a distinct plateau region where the mechanical moduli remained nearly constant in the range 0.01–5 Pa (Figure 4). The observed plateau likely reflects the formation of physical interactions between biopolymer chains, resulting in the formation of a three-dimensional network structure [39]. However, the mechanical moduli of the tested nanoemulsions (AM, CO, and AH), particularly G′, were slightly lower than those of the control sample, confirming differences in rheological properties.

These observations are consistent with findings previously described in the scientific literature. The observed changes result not only from the applied additives but also from the homogenization process used during nanoemulsion preparation [18]. Beyond the LVR, a rapid decrease in the values of G′ and G″ was observed with increasing strain, indicating progressive disruption of the internal molecular organization of the samples (Figure 4). The influence of extract concentration in the emulsion was not clearly evident. A slight decrease in the modulus at higher extract concentrations was observed only for the AM sample (Figure 4a).

**Figure 5:**
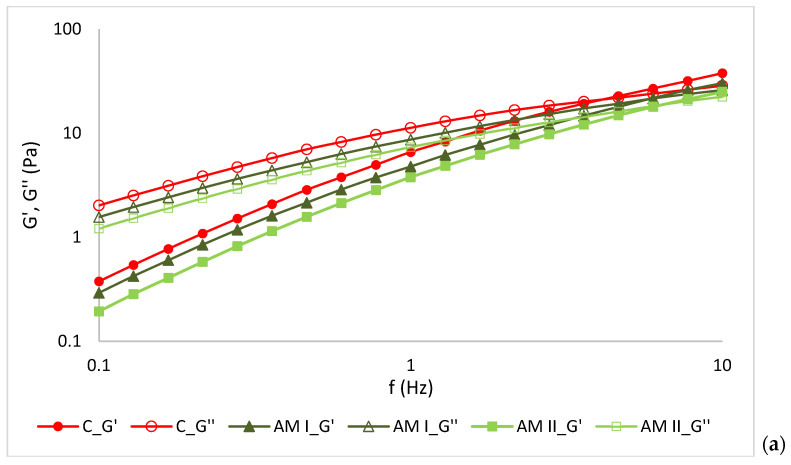

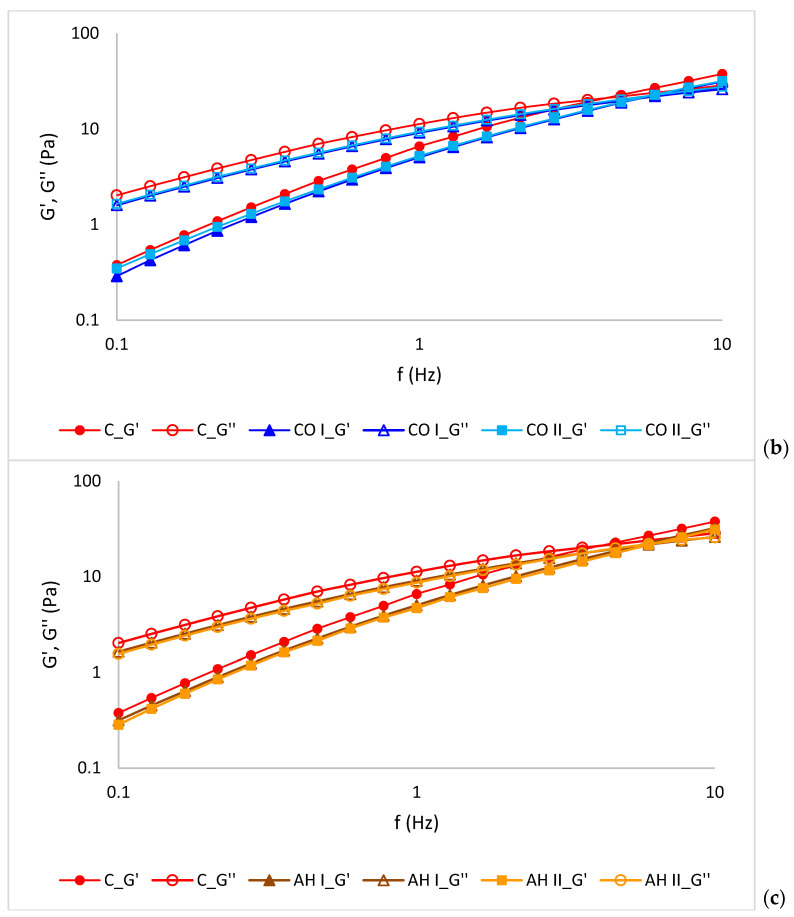
Values of storage and loss moduli (G′ and G″) measured in frequency sweep test of (**a**) AM, (**b**) CO and (**c**) AH.

At small deformations, the dominance of the loss modulus over the storage modulus (G′ < G″) in the lower value of frequency range (Figure 5), confirmed the viscous-like character of the emulsions. A clear crossover between the modules was observed in the upper frequency range. At low oscillation frequencies hyaluronan macromolecules have sufficient time to reorganize and return to their original conformation through the gradual disentanglement of polymer chains, resulting in predominantly viscous behavior. In contrast, at high frequencies corresponding to rapid deformation, the entangled polymer network responds quickly by forming new intermolecular interactions and entanglements, which leads to a more elastic material response [17,40]. A similar trend was observed for hyaluronic acid-based emulsions enriched with plant extracts. However, a slight reduction in both mechanical moduli was recorded compared with the pure hyaluronic gel, and the crossover between the viscous and elastic moduli occurred at higher frequency values. This behavior may be associated with weaker intermolecular interactions and reduced hydrogen-bond-mediated molecular association in the modified systems. Other authors have reported that the mechanical properties of hyaluronic acid gels depend strongly on their molecular weight [41]. Venerová and Pekař [42] observed that in low-molecular-weight gels, viscous behavior predominates (G′ < G″). As the molecular weight increases above approximately 800 kDa, a crossover of the moduli can be observed. At around 2 MDa, hyaluronic acid begins to exhibit predominantly elastic behavior [42]. This phenomenon has been explained by increased molecular association resulting from hydrogen-bond interactions between polymer chains [40]. Grabowski et al. [43] as well as Di Meo et al. [44] also confirmed that the development and stability of the HA network are governed by both molecular weight and concentration. HA gels prepared from low-molecular-weight polymer showed Newtonian flow behavior, suggesting only limited intermolecular entanglement, which may be attributed to the relatively rigid, rod-like conformation of the chains. This structural organization was highly resistant to both thermal sterilization and enzymatic hydrolysis by hyaluronidase. Conversely, HA of higher molecular weight exhibited lower stability against thermal treatment and enzymatic hydrolysis, while the degree of depolymerization increased with increasing molecular weight and diminished with rising concentration.

From an application standpoint, this indicates that the developed wound dressings will exhibit predominantly viscous behavior at low deformations, which may facilitate spreading on the skin. At higher deformations, however, they will demonstrate a more elastic response, supporting the maintenance of structural integrity after application. The slight reduction in mechanical moduli observed in extract-enriched systems may further enhance application comfort without significantly compromising structural stability.

### 3.6. Texture Properties of Emulsions

**Figure 6:**
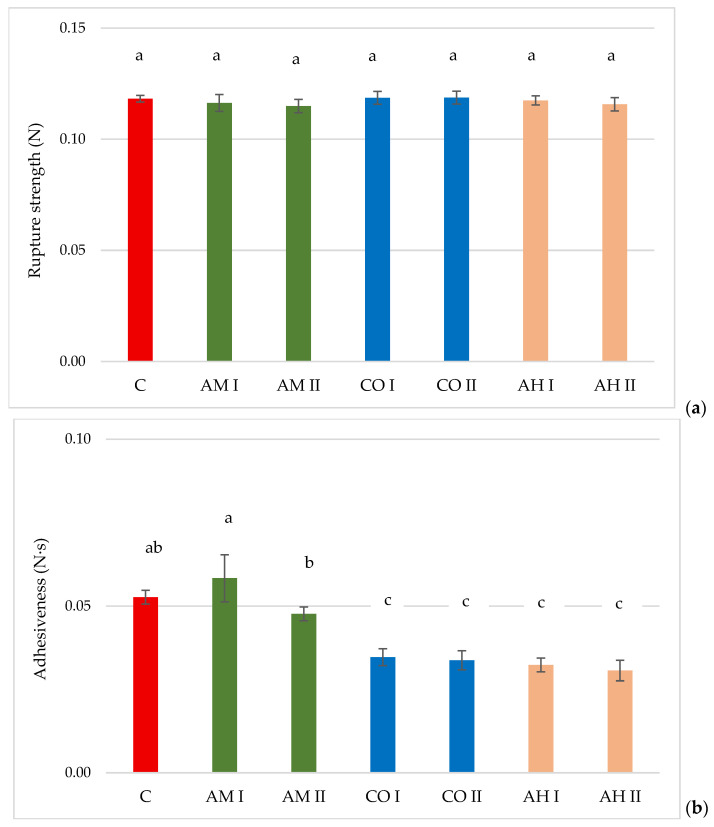
Textural parameters of emulsions. Sample C designates the control sample (i.e., H0). (**a**) Rupture strength of the samples, (**b**) adhesiveness of the samples. The presence of the same superscript letter (**a**–**c**) indicates that there is no statistically significant difference between the values (*p* < 0.05).

The rupture strength and adhesiveness of the tested emulsions are presented in Figure 6. The maximum force recorded during sample penetration, corresponding to the point of gel structure failure (rupture strength), was within a similar range for all analyzed emulsions, as confirmed by statistical analysis (Figure 6a). The incorporation of plant extracts did not weaken the structure of the hyaluronic acid gel network. Differences, however, were observed in the adhesiveness values of the samples (Figure 6b). The adhesive behavior of topical formulations is determined by two primary mechanisms: chemical interactions and physical adhesion [45]. Chemical adhesion arises from intermolecular forces such as ionic, covalent, and metallic bonds, as well as hydrogen bonding and van der Waals interactions. In contrast, physical adhesion is mainly associated with mechanical interlocking and topological interactions between the formulation and the contact surface [46]. The addition of arnica extract had the smallest effect, and the difference was not statistically significant. In contrast, calendula and horse chestnut extracts reduced the adhesiveness of the samples by up to 40% compared with the control. Lower adhesiveness of the emulsions may, on the one hand, facilitate spreading of the formulation on the skin, but on the other hand, it may shorten the contact time of active compounds, which in the case of medicinal ointments may be an undesirable effect [47]. However, it should be emphasized that the final technological suitability of emulsions containing extracts is determined by multiple factors discussed in this study and depends on achieving a balance between adequate structural strength (maintained in all samples), appropriate rheological performance, and an optimal level of adhesiveness that ensures both ease of application and therapeutic effectiveness.

### 3.7. Preliminary Screening of Plant-Based Emulsions and Reference Antiseptics

As a preliminary test, antimicrobial activity of nanoemulsions containing arnica, calendula and chestnut extracts, was evaluated against 57 microbial isolates, including 32 Gram-positive bacteria, 13 Gram-negative bacteria and 12 yeasts. Due to their high viscosity, all emulsions were tested using the well diffusion method. For comparison, four commercially available antiseptic preparations (stye eye lipogel, surface disinfectant, skin disinfectant and hydrogen peroxide) were examined using disk diffusion.

When focusing on the plant-derived formulations, arnica-based emulsions (AM1 and AM2) caused the largest growth inhibition of bacteria (particularly against Gram-positive cocci) (Table 4). Horse chestnut showed weak to moderate antimicrobial activity (the higher chestnut extract concentration caused the largest growth inhibition of yeasts), while the activity of calendula was near to negligible. Hyaluronan control did not inhibit the growth of microorganisms.

As expected, all antiseptic preparations demonstrated broad-spectrum antimicrobial activity, resulting in large growth inhibition zones across nearly all bacterial and yeasts isolates. Stye eye lipogel was the least effective among the examined antiseptics.

These results pointed to the selective activity of the plant-based emulsions. This preliminary screening clearly indicated that arnica extract was the most effective in bacterial growth inhibitions, and as such was selected for further tests, conducted using an expanded microbial panel.

Based on the preliminary findings, emulsions containing two concentrations of arnica extracts (AI and AII) were tested against 110 microbial isolates, including 76 Gram-positive bacteria, 19 Gram-negative bacteria and 15 yeasts commonly associated with healthy skin microbiota and diseased human skin. The experiment was conducted using the well diffusion method.

Across the entire group of microorganisms tested, the arnica-containing emulsions exhibited the highest antimicrobial activity against Gram-positive bacteria (Figure 1). This was most clearly visible in the case of the genus *Staphylococcus*, within which most isolates reacted to both arnica extract concentrations, with AII mean growth inhibition zones exceeding 20 mm in the case of *S. hominis*, and methicillin-resistant *S. aureus*, *S. capitis* and *S. petrasii* (20.5, 23, 30 and 30 mm, respectively). A clearly smaller growth inhibition was observed in the case of AI, indicating an evident concentration-dependent effect of these formulations (Table 5). Apart from *Staphylococci*, *Rothia mucilaginosa* also showed high susceptibility to the arnica-based formulations (growth inhibition zone of 25 and 38 mm for AI and AII, respectively).

In contrast, Gram-negative bacteria were mainly resistant to both arnica formulations. These included MBL-positive *Klebsiella pneumoniae*, *Pseudomonas aeruginosa*, *Pantoea agglomerans* and *Pseudescherichia vulneris*. The growth of *Enterobacter kobei*, *Escherichia coli*, *Pseudomonas stutzerii* and *Serratia marcescens* was inhibited by both concentrations of arnica extracts but these inhibitions were much smaller than those recorded for most Gram-positive isolates.

**Figure 7:**
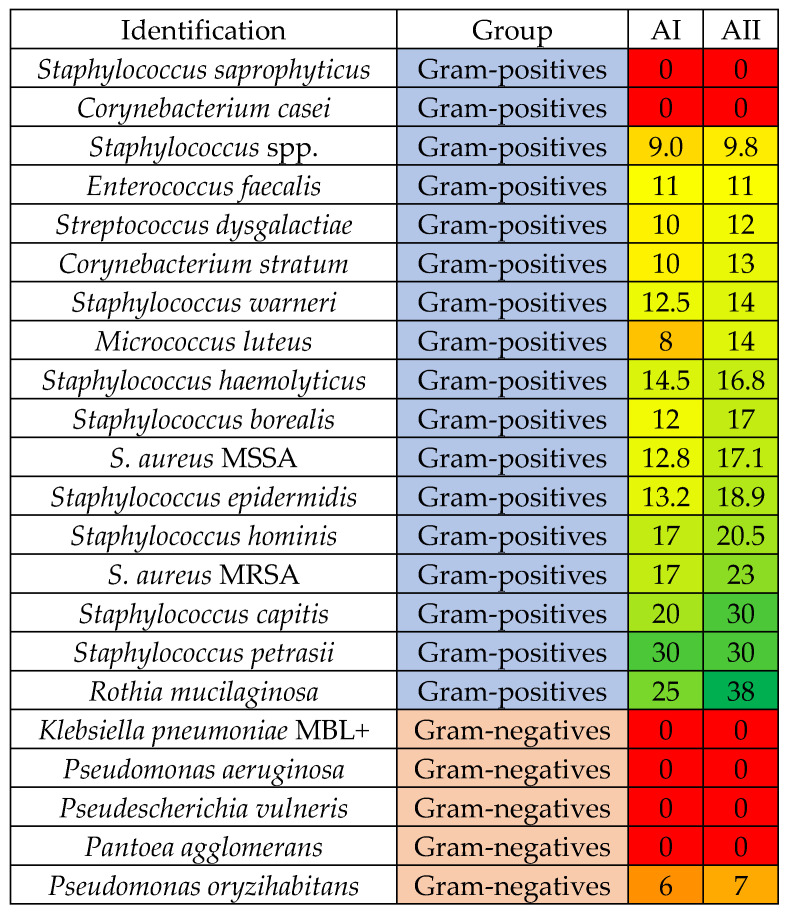

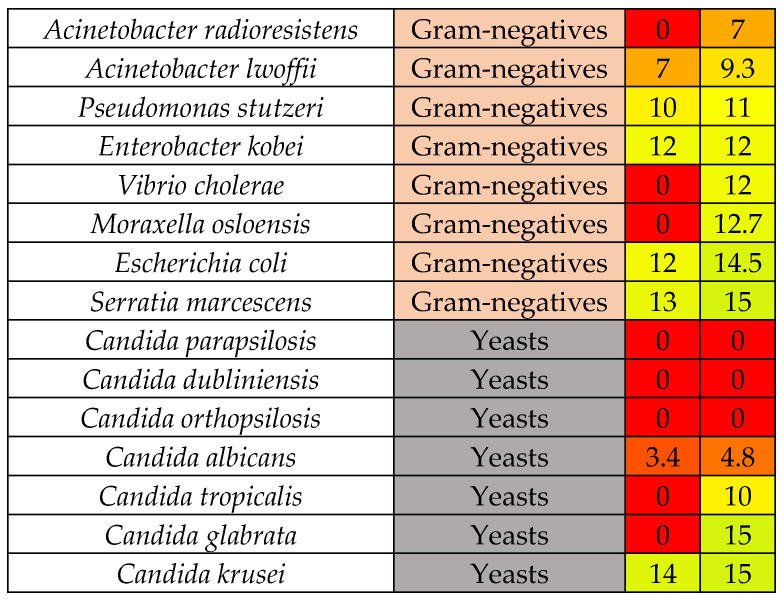
Gantt chart showing the species-specific activity of nanoemulsions containing two concentrations of arnica extracts (AI and AII). It presents the mean growth inhibition of Gram-positive bacteria, Gram-negative bacteria and *Candida* yeasts exposed to arnica extract-based nanocomposites. Groups of microorganisms are color-coded in the central column (blue for Gram-positive, pink for Gram-negative and gray for yeasts). Growth inhibition values are shown using a heat scale: red indicates no inhibition (mean growth inhibition of 0 mm), green represents the strongest growth inhibition (the largest inhibition zones), and intermediate colors reflect a scale of intermediate inhibition zones.

Finally, only a few yeast isolates reacted to the application of arnica-based formulations, with *C. albicans* and *C. krusei* as the only ones whose growth was inhibited by both AM1 and AM2 (Figure 7).

The microbiological part of the study demonstrated that the developed formulations containing plant-derived extracts differed significantly in terms of their antimicrobial activities. These differences open distinct possibilities of their potential dermatological applications. The observed plant extract-dependent differences in antimicrobial activity are consistent with previously reported properties of the three plants examined in this study. Although these differences suggest that phytochemical composition plays an important role in shaping the antimicrobial activity, the present study did not include a compositional analysis of the three extracts, which constitutes a limitation of our experiments. Nevertheless, the available literature provides valuable insight into the groups of compounds probably responsible for the observed effects. Extracts of *Arnica montana* are rich in sesquiterpene lactones (primarily helenalin and dihydrohelenalin), phenolic acids and flavonoids, associated with antibacterial activity [48]. *Calendula officinalis*, on the other hand, contains high levels of carotenoids, triterpenoids, and saponins, the major activity of which is anti-inflammatory and wound healing [49]. Finally, flowers of *Aesculus hippocastanum* are a rich sources of carotenoids, flavonoids (kaempferol, quercetin, astragalin) and small amounts of coumarins [50]. Studies on antimicrobial activity of *A. hyppocastanum* are rather limited and they demonstrate a rather weak antibacterial and antifungal activity of horse chestnut extracts with high MIC values [50]. The strongest inhibitory effects were observed for the arnica-based nanocomposites, whereas calendula-based composites showed minimal antimicrobial activity, and chestnut-based formulations exhibited selective antifungal activity primarily directed toward *Candida* spp. The antimicrobial efficacy of arnica extracts aligns with previous literature reports, demonstrating that *A. montana* extracts exert antimicrobial and antibiofilm activity against Gram-positive bacteria, e.g., *Staphylococcus* spp., including MRSA, *Streptococcus mutans*, and *Enterococcus faecalis*, as well as against Gram-negative bacteria (*Escherichia coli*) and—to a lesser extent—against *Candida albicans* [51,52]. On the other hand, formulations containing calendula extract resulted in weak antibacterial effects, with only a few Gram-positive strains inhibited. Our obsevarion is contrary to numerous reports showing the broad antimicrobial activity of *C. officinalis* extracts [53]. However, polyphenols—a group of compounds present in calendula extracts—have been described to have a positive effect on probiotic microorganims [53]. Calendula extracts have also been suggested to modulate the composition of skin microbiota by changing it towards the decrease in taxa involved in skin inflammations and enrichment of taxa with the immunomodulatory properties of the Bacillaceae family [54]. Horse chestnut–based formulations showed moderate activity, with inhibitory effects more visible in the case of *Candida* yeasts. Our observations are in line with literature reports on the limited antibacterial activity of *A. hippocastanum* extracts against Gram-negative and Gram-positive bacteria [55] coupled with the reproted antifungal activity against not only *Candida*, but also *Aspergillus*, *Penicillium* and *Mucor* sp. [56]. Given that *Candida* species are common skin and mucosal opportunists, these results highlight the potential of horse chestnut extract as a candidate for formulations targeting mild superficial fungal colonization or aiming to prevent yeast overgrowth while maintaining microbiota balance.

The differences in microbial susceptibility to the examined formulations can be explained by the known structural and physiological characteristics. Gram-positive bacteria, with their thick peptidoglycan layer that more easily absorbs various chemical agents, and thus accessible cell wall components, are generally more susceptible to plant-derived phenolics, terpenoids, and flavonoids than Gram-negative bacteria whose outer membrane containing phospholipids and lipopolysaccharides acts as a barrier to antimicrobial agents [57]. In our study, the group of Gram-positive bacteria, comprising mostly *Staphylococccus* spp., exhibited the highest susceptibility profiles to all examined compounds, including disinfectants.

Finally, the differential antimicrobial profiles of the three extract-based nanocomposites suggest that they might become suitable for distinct potential applications. And so, calendula-based formulations with minimal antimicrobial activity, which exert little to no suppression of commensal skin bacteria or yeasts, may be particularly well suited for microbiota-safe cosmetic and dermatological preparations. Their well-documented wound-healing, anti-inflammatory, and antioxidant properties can support skin regeneration without disturbing the beneficial skin flora, aligning with the growing interest in microbiome-friendly skincare. This is supported by the literature emphasizing calendula’s therapeutic benefits without antimicrobial activity, including roles in tissue repair and the modulation of inflammatory pathways [34,53,54]. The chestnut-containing composites characterized by selective activity against *Candida* spp., could be valuable for targeted antifungal support, for example in formulations intended for intertriginous areas prone to yeast overgrowth or as adjuncts in gentle antifungal skincare. Their moderate activity profile suggests they may help control fungal opportunists without broadly suppressing bacterial members of the microbiota [55,56]. Finally, formulations containing nanocapsules of *Arnica montana* extracts, given their robust activity (particularly against *Staphylococcus* species, including MRSA) represent promising candidates for topical preparations used after aesthetic medicine procedures, microneedling, injections, laser therapy, or minor skin injuries, where transient breaches in the epidermal barrier create opportunities for bacterial invasion. The strong anti-staphylococcal and antibiofilm activity described in previous studies [8,36,51] suggests potential usefulness in reducing colonization by opportunistic pathogens in compromised skin environments. Such preparations may also find application in burn care, post-surgical sites, or dermatological treatments where localized antimicrobial protection is desirable.

## 4. Conclusions

This study demonstrates that hyaluronic acid-based nanocomposites enriched with plant extracts of *Arnica montana*, *Aesculus hippocastanum*, and *Calendula officinalis* can be successfully formulated using a combined sonication–homogenization approach. Structural analyses confirmed the formation of stable spherical nano/microcapsules embedded within the HA matrix, while FTIR and UV-Vis spectroscopy indicated effective incorporation of both the oil phase and extract-specific phytochemicals without compromising their chemical integrity.

The color intensity and hue of the emulsions will shape the visual perception of the resulting wound dressings, with warmer tones suggesting higher bioactivity, green shades conveying naturalness, and greater saturation enhancing both aesthetic appeal and perceived efficacy. Rheological and texture measurements demonstrated that all tested emulsions maintained structural integrity. Nevertheless, their practical applicability depends on achieving an appropriate equilibrium between mechanical durability, viscoelastic properties, and a level of adhesion that provides convenient spreading while preserving effective therapeutic performance.

Despite similarities in physicochemical performance, the three extract types displayed clearly different antimicrobial effects. Arnica-containing formulations exhibited the strongest and broadest antimicrobial activity, particularly toward Gram-positive members of the skin microbiota, demonstrating a clear concentration-dependent effect. Chestnut-based formulations showed selective antimycotic activity, especially against *Candida* species, while exerting only minor antibacterial effects. Antimicrobial activity of calendula-based composites was very limited, indicating that their biological usefulness is likely attributed to non-antimicrobial properties such as anti-inflammatory or wound-healing potential.

These distinct antimicrobial effects suggests that the HA-based bionanocomposites described in our study may be used in a variety of applications. The microbiota-neutral calendula formulations may be most suitable for cosmetic or dermatological products designed to support skin homeostasis without disrupting the commensal microbiota. Chestnut composites may serve in formulations where mild antifungal activity is desired. Finally, arnica-based bionanocomposites are promising substances that could be used in topical preparations intended for use when delicate antibacterial activity is beneficial, e.g., after cosmetic or aesthetic medicine procedures, as well as after minor skin injuries.

The ongoing cytotoxicity and genotoxicity assessments of the developed bionanocomposites will provide further information on the applicability and future consumer-safety.

## Figures and Tables

**Table 1:** Extraction yield of plant extracts used in nanocomposite formulations.

Raw Material	Extraction Yield (%)
*Arnica montana*	49.02
*Calendula officinalis*	44.25
*Aesculus hippocastanum*	42.38

**Table 2:** Color parameters of emulsions.

Samples	L* (D65)	a* (D65)	b* (D65)	C*	h*	∆E*
H0	97.00 ± 0.55 ^a^	0.06 ± 0.03 ^f^	0.97 ± 0.05 ^g^	0.97 ± 0.05 ^g^	1.51 ± 0.03 ^c^	-
AM I	57.30 ± 0.02 ^e^	−0.66 ± 0.01 ^g^	28.35 ± 0.04 ^d^	28.36 ± 0.04 ^c^	178.45 ± 0.00 ^a^	48.23 ± 0.04
AM II	45.26 ± 0.06 ^f^	2.71 ± 0.01 ^b^	28.83 ± 0.06 ^c^	16.77 ± 0.02 ^f^	1.55 ± 0.00 ^b^	34.99 ± 0.01
CO I	67.45 ± 0.01 ^b^	0.80 ± 0.01 ^d^	29.67 ± 0.05 ^b^	29.68 ± 0.05 ^b^	1.54 ± 0.00 ^b^	41.20 ± 0.03
CO II	65.03 ± 0.45 ^d^	3.50 ± 0.02 ^a^	34.79 ± 0.19 ^a^	34.97 ± 0.19 ^a^	1.47 ± 0.00 ^e^	46.67 ± 0.30
AH I	65.78 ± 0.02 ^c^	0.41 ± 0.01 ^e^	16.67 ± 0.32 ^f^	16.77 ± 0.02 ^e^	1.55 ± 0.00 ^b^	18.31 ± 0.02
AH II	67.56 ± 0.03 ^b^	1.58 ± 0.01 ^c^	19.45 ± 0.07 ^e^	19.52 ± 0.07 ^d^	1.49 ± 0.00 ^d^	15.66 ± 0.07

The values are expressed as the mean *±* standard deviation. The presence of the same superscript letter (a–g) in each column indicates that there is no statistically significant difference between the values (*p* < 0.05).

**Table 3:** Illustrative color of the tested emulsions.

	H0		AM I	AM II		CO I	CO II		AH I	AH II	
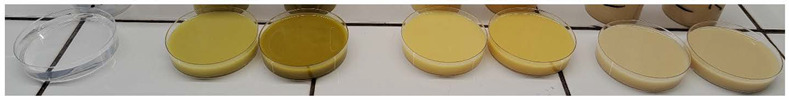											

**Table 4:** Mean inhibition zones observed for each preparation across Gram-positive and Gram-negative bacteria, and yeasts. The results are presented as means ± standard deviations. Bolded values show the largest growth inhibition zones.

Preparation	Gram-Positive	Gram-Negative	Yeasts
Arnica (I)	15.50 (6.9)	3.31 (4.6)	0.50 (1.7)
Arnica (II)	**21.81 (10.9)**	**8.46 (5.3)**	5.25 (9.8)
Calendula (I)	1.53 (3.7)	0.69 (2.5)	0.50 (1.7)
Calendula (II)	2.38 (5.2)	0 (0)	5.17 (9.3)
Chestnut (I)	6.50 (7.2)	0 (0)	6.50 (8.9)
Chestnut (II)	10.50 (8.3)	0.77 (2.8)	**11.08 (10.7)**
Control (H0)	0 (0)	0 (0)	0 (0)
Stye eye lipogel	17.41 (20.2)	0 (0)	4.17 (9.9)
Surface disinfectant	36.00 (16.3)	15.38 (7.2)	24.00 (19.8)
Skin disinfectant	44.13 (10.0)	23.77 (3.8)	**46.75 (15.3)**
Hydrogen peroxide	**53.75 (7.29)**	**42.77 (12.9)**	43.25 (19.6)

**Table 5:** Susceptibility of the three examined microbial groups (Gram-positive bacteria, Gram-negative bacteria and *Candida* yeasts) to two concentrations of arnica extracts (AMI and AMII). The values presented in the table are means and median values of the recorded growth inhibitions zones (mm).

Microbial Group (*n*)	Parameter	AI	AII	Control (H0)
Gram-positives (*n* = 76)	mean	11.96	15.30	0
	median	12	14	0
	SD	7.22	10.35	0
Gram-negatives (*n* = 19)	mean	4.84	8.74	0
	median	0	10	0
	SD	5.52	5.81	0
*Candida* yeasts (*n* = 15)	mean	2.07	5.93	0
	median	0	0	0
	SD	4.54	9.27	0

## Data Availability

The original contributions presented in this study are included in the article. Further inquiries can be directed to the corresponding author.
